# Sustainable Working Life in Intensive Care: A Qualitative Study of Older Nurses

**DOI:** 10.3390/ijerph19106130

**Published:** 2022-05-18

**Authors:** Marta Sousa-Ribeiro, Petra Lindfors, Katinka Knudsen

**Affiliations:** Department of Psychology, Stockholm University, 11 419 Stockholm, Sweden; pls@psychology.su.se (P.L.); katinkaknudsen@hotmail.com (K.K.)

**Keywords:** retirement decisions, extended working lives, older nurses, intensive care, SwAge model, interpretative phenomenological analysis, qualitative

## Abstract

To counteract the shortage of nurses in the workforce, healthcare organizations must encourage experienced nurses to extend their working lives. Intensive care (IC) has higher nurse-to-patient ratios than other settings, which includes a particular susceptibility to staff shortage. This qualitative study investigated how older IC nurses experienced their working life and their reflections on the late-career and retirement. Semi-structured interviews with 12 IC nurses in Sweden (aged 55–65 years) were analyzed using an interpretative phenomenological analysis approach. The results showed that nurses planned to continue working until the age of 65 and beyond. When reflecting on their late-career decisions, nurses considered nine areas covering individual, work, and organizational factors as being central to their ability and willingness to stay. Overall, the nurses had good health and were very satisfied and committed to their job and to the organization. They mentioned having both the job and personal resources required to cope with the physical and mental job demands, which were perceived as motivational challenges, rather than hinders. They also reflected on various human resource management practices that may promote aging-in-workplace. These findings may inform organizations aiming at providing adequate conditions for enabling healthy and sustainable working lives for IC nurses.

## 1. Introduction

During the last decades, many industrialized countries have seen the number and proportion of the population aged 65 years or older increase rapidly [1]. In Sweden, women and men aged 65 in 2018 would expect to live an additional average of respectively 22 and 19 years (for both women and men, 16 of these years were to involve no severe or moderate health problems) [2]. Moreover, in Sweden, the number of people aged 80 years or older is expected to increase by 50 percent by 2029 [3]. These changes in the age structures and demographic aging have profound implications at various levels and threaten the sustainability of healthcare, long-term care, and pension systems [1,2].

To respond to the challenges associated with the aging of the population, several European Union member states have implemented substantial reforms to their pension systems. Mainly, these reforms have focused on increasing the age of effective retirement and on tightening the eligibility requirements to qualify for retirement, with stronger disincentives to early retirement and advantages for postponed retirement [4]. These reforms have had an effect on the labor market participation of older workers (aged 55–64 years) in the European Union, with employment rates increasing from 38% in 2000 to 62% in 2019 [4]. 

The Swedish pension system is flexible and allows employees to work until a later age. In Sweden, there is no statutory retirement age, and employees have the possibility to retire between ages 62 and 68 (with financial benefits for later retirement), and most have a possibility to work beyond age 68 if the employer agrees [5]. For an increasing number of people, retirement decisions now mostly relate to decisions about when to start drawing a pension. Instead of an abrupt transition out of the workforce, retirement may involve a gradual process of labor market withdrawal. The pension can be drawn as 25–100 percent of the whole, and besides being allowed to suspend and subsequently restart the payment of the pension at any time, the retirees may continue to work and earn new pension entitlements after having started to draw their pensions [6]. Sweden has a relatively high employment rate of workers aged 60–64 (70%), but this rate decreases to 24% among workers aged 65–69 [7]. In 2019, the average age of starting to take up the retirement pension was 64.6 years. Although there is a relatively late-exit culture in Sweden, as compared with other countries [8], preferences for early retirement seem common. In a survey conducted in 2015 [2], the average age until which employees thought that they would be able to work in their current job or a similar one was 68.0 years for men and 67.1 years for women, whereas the average ages people wanted to work to were 63.3 years (men) and 62.8 years (women), respectively. Thus, many still prefer to retire much earlier than they probably can and will [9].

An aging workforce adds challenges to organizations which face shortages of qualified workers. Healthcare is a key sector that is particularly pressured by the increase in life expectancy and the number of older people, with associated increases in chronic diseases as well as healthcare needs and demands [1]. Nurses are key in healthcare organizations and are the largest group of health and social care workers in many OECD (Organisation for Economic Co-operation and Development) countries, corresponding to approximately 20–25% of all workers [1]. The age composition of nurses follows that of the general population [1] and is aging as well. In Sweden, the age distribution of nurses is relatively even, but the 50–54-year-olds constitute the largest group [10]. In 2020, the average retirement age was 63.8 years, with 5.8% of the nurses being aged 65 and older including those who postponed retirement and those who worked on an hourly basis after having started to receive their pension benefits [11].

In Sweden, as elsewhere, there is an enormous lack of qualified nurses [1,10,12]. In 2018, Swedish healthcare providers reported facing shortages of general and specialist registered nurses in all areas, including anesthesia, intensive care, and surgical care, which are key areas where 80 percent of the organizations reported a lack of staff [10]. This obviously challenges the capability to provide efficient and high-quality healthcare, with staff shortage also adding stress [13]. To address the shortage of nurses in an ever-increasing competitive labor market searching for registered nurses, healthcare organizations need to attract new staff and motivate their experienced nurses to postpone the point of fully retiring from the nursing profession [12]. For example, a report from the Swedish Association of Local Authorities and Regions in 2017 [14] stated that the recruitment needs of the Swedish welfare, including healthcare, until 2026, would decrease by 50,000 individuals by postponing the average retirement age by two years. Besides alleviating the staff shortage, older nurses who extend their working lives contribute to the organization with the experience, proficiency, and wisdom that they have acquired over the years [13,15,16]. This is important for the quality of the care provided as well as for the guidance of entry-level nurses [17]. In view of this, it is both timely and important to investigate how older nurses experience their working life and describe the factors that are important when considering a late career and whether to retire or not [18]. Such knowledge may be important for organizations and policymakers to design policies and practices enabling and motivating nursing staff to remain in the workforce [19]. 

An aging workforce is a global phenomenon, and retirement has turned into an important research topic. An increasing number of research literature has investigated older workers’ late-career and retirement decisions (for meta-analyses and literature reviews, see [20,21,22,23]). Wang and Shi [24] systematized the factors that influence the retirement process into four categories: (1) individual attributes (e.g., demographic characteristics, health, and financial circumstances), (2) family factors (e.g., marital and dependent care status, and spouse’s working status), (3) job and organizational factors (e.g., job characteristics, job attitudes, and age stereotypes at work), and (4) socioeconomic factors (e.g., social norms about retirement, and the social security system). Moreover, Browne et al. [25] focused their systematic review on the relationships between workplace psychosocial environments, retirement intentions, and actual retirement, and found that high job satisfaction and high job control were associated with later retirement intentions and actual retirement. However, occupations within human services, such as nursing, involve direct contact with people and are characterized by fewer opportunities for control and planning than occupations in other sectors [26].

A number of studies have investigated factors associated with nurses’ retention [19,27], intention to remain [28,29,30], intention to leave [31,32,33,34,35,36], turnover [19,37,38], retirement planning [39,40], retirement preferences [16,18,41,42,43,44,45,46,47,48,49], and post-retirement work [50]. Although turnover and retirement constitute distinct types of organizational withdrawal, there are similarities [51], and all these outcomes are of interest when investigating older nurses’ late-career decisions. 

Research has found that there are several individual-related factors involved in older nurses’ decisions regarding whether or not to remain in the workforce, including health status and work ability [16,43,46,48,49], financial motives [27,43,46,48,49], social motives [27,45,49], and family situation and leisure time [16,43,46,48]. The psychosocial work environment has also been found to impact significantly on older nurses’ late-career plans [16,18], and a number of work-related factors have been found to be important, such as workload [46,48], work pace [46], autonomy and control [46], role clarity [30], flexible working conditions, flexible working schedules and work-time control [27,30,48], shift work [48], recovery opportunities [46], opportunities for competencies development [41,45,48], recognition [28,48], leadership style and supervisor support [16,30,48], challenging, varied, and interesting work [27,45], meaningful work [45], and job satisfaction [16,45]. 

Nursing work is often experienced as physically and mentally demanding [19,43,48], and has consistently been associated with poor health including musculoskeletal disorders, high levels of work-related stress, and burnout, as well as with job dissatisfaction [17,48,52]. Due to the particularly fast-paced work, excessive workload, limited decision authority, long work shifts, demands of continuous and close monitoring of severely ill and unstable patients, the need of handling sophisticated life support equipment, the frequent exposure to critical and traumatic events, and confrontation with ethical dilemmas, nursing work in intensive care (IC) units is associated with higher levels of physical and emotional demands, work stress, burnout, and depression as compared with other healthcare environments [36,53,54,55]. Furthermore, IC units require a higher nurse-to-patient ratio than other healthcare settings—in Sweden, this is normally 1:1–2 [56]. This means that IC units are particularly vulnerable to the shortage of nursing staff [36], something that has been aggravated during the COVID-19 pandemic [56]. However, despite the increasing importance of IC nurses to the sustainability of the healthcare sector, the characteristics of older IC nurses’ late-career decisions seem understudied.

### The Present Study

This study investigated how older IC nurses experienced working life and reflected on their late-careers and retirement. Using a qualitative interpretative phenomenological approach (IPA, [57,58]), we aimed to add to previous research findings and to provide a fine-grained perspective and new insights into IC nurses’ sensemaking of their experiences of working and approaching retirement in IC, and their thoughts concerning what may influence their late-career decisions. The study findings may be important for organizations and policymakers in the design of policies and practices targeted at enabling healthy and sustainable working lives, and motivating experienced IC nursing staff to remain in the workforce longer. 

## 2. Materials and Methods

### 2.1. Participants and Procedure

The study was part of a research project investigating older workers’ retirement decision making and was approved by the Swedish Ethical Review Authority (ref no. 2017/1720-31/5). The recruitment of interviewees involved purposeful sampling of nurses in an IC unit in a general hospital in a large city in Sweden, which employed approximately 85 nurses, 26 of those aged 55 years or older, and followed three criteria: (1) working as a specialized nurse in the IC unit; (2) aged 55 years or older; and (3) not yet retired. 

The third author contacted the head of the IC unit and the operational manager and presented the study aim and the sampling criteria. The managers granted permission for the implementation of the study at the unit, and a leaflet with information about the research project and contact details to the research team was distributed to the nurses. Those interested in participating contacted the team, received a brief description of the study again, and then provided written informed consent. 

The participants included 12 IC nurses aged between 55 to 65 years who specialized in either IC or anesthesia. Four participants worked full time, while the others were working part time (ranging between 50% and 90% of a full-time schedule). Besides their clinical work within the IC unit, five nurses had other roles in the hospital, such as work in the emergency room, training and supervision of students, and IT and technical support of clinical devices. Organizational tenure ranged between 2 and 36 years; the nurses with the shortest tenures had long experiences of working as nurses elsewhere. All but four interviewees were women, which to some extent reflects the gender distribution of the nursing occupation in Sweden [26]. The relatively small and homogeneous sample of 12 IC nurses aligns with the IPA approach adopted in this study, which typically includes smaller samples ranging from 5 to 10 participants [58]. This allowed for a detailed and nuanced analysis, as well as for some variation in the subjective experiences while keeping with the ideographic focus characterizing the IPA approach. 

### 2.2. Interviews

The semi-structured interviews took place in May–June 2019, in a private room at the hospital during working time, and lasted around 40–65 min. All interviews were audio-recorded and conducted in Swedish by the third author. The interviews were transcribed verbatim by the third author, with all names and other details that would somehow identify interviewees being masked during the process, to maintain interviewee integrity and to follow research ethics. The transcripts’ length ranged between 4865 and 10,364 words (median 7363 words). 

The interview guide covered the following main topics: (1) experience of working as an IC nurse at the organization; (2) perceptions of aging in the workplace, (3) attitudes toward work and retirement, and (4) thoughts and preferences regarding the retirement transition. The questions were open-ended and the interviewees were encouraged to expand freely on the topic. Example questions were as follows: “How do you experience your work as an IC nurse at this unit?”, “What place has work in your life?”, and “What are your thoughts on retirement?”. Potential prompts were prepared to facilitate the flow of the interview. The interview guide was first pilot tested with two older nurses, which resulted in minor language adjustments in order to improve the clarity of the questions and ensure the validity of the interview guide. 

### 2.3. Analytic Approach

The methodological approach used in this study was primarily the IPA [57]. The IPA was chosen due to us being interested in exploring the nurses’ sensemaking of their experiences of working in IC, and of approaching retirement. Specifically, the use of IPA allows for an in-depth exploration of subjective experiences in a particular context [57]. It involves a so-called double hermeneutic, as the researcher aims at interpreting and making sense of the participants’ sensemaking of their own lived experiences [57]. The use of IPA follows an inductive approach to interpret interview data, departing from the participants’ narratives and then forming theoretically driven interpretations of the meanings identified in the transcripts. 

The analyses of the transcripts were conducted separately by the first and third authors. This was followed by a discussion around the possible interpretations of the extracts and a construction of a thematic structure that increased the validity of the study findings. At first, the analytical process followed the heuristic framework proposed by Smith [57] for analyzing qualitative data using the IPA approach. In brief, each transcript was carefully read and re-read a few times, with initial notes being made on the extracts that were to be considered of interest for the study. Some of these notes were purely descriptive, while others were more conceptual and interpretative. Then, the analyses involved establishing associations and patterns of meaning across the initial notes within each transcript. This allowed for the identification of emergent themes, which reflect both the interviewee’s narrative and the researcher’s interpretations while keeping an ideographic focus on the individual voices. This process was followed by the development of an initial structure, built on the relationships across the emergent themes that were considered relevant for the study aim. This, in turn, resulted in the development of higher-level “superordinate themes”, which clustered together a number of related “subordinate themes”, and represented a higher concept of meaning. Finally, the analyses focused on identifying commonalities and convergences, as well as nuances and divergences across the different transcripts, and the themes were retained on the basis of their meaningfulness in representing the subjective meanings of the experiences of the interviewees and the relevance of the theme for the study aim. In the final phase, this inductive and data-driven analytic approach, which identified themes emerging from the narratives, was combined with a more deductive approach, which was informed by the SwAge model (sustainable working life for all ages) [59], a theoretical model for sustainable working life for all ages that proposes nine areas to be determinant to the individual decisions of whether to extend the working life or to retire. This combination of the inductive and deductive approaches contributed to refining the thematic structure and guided the final labeling of the superordinate or higher-order themes.

## 3. Results

The analysis generated ten superordinate themes, the first centering on the nurses’ retirement decision making, and nine themes that reflect the nine determinant areas proposed by the SwAge model [59] to be involved in the individual’s decision of whether to extend the working life or to retire: (1) self-rated health, diagnoses, and functional diversity; (2) physical work environment; (3) mental work environment; (4) work schedule, work pace, and recovery time; (5) personal finances; (6) personal social environment and private life; (7) social work environment, discrimination, leadership style, and age management; (8) motivation, satisfaction, and stimulation in the execution of work tasks; and (9) competence, use of skills, knowledge, and opportunities for development at work (see Table 1 for an overview).

### 3.1. Retirement Decision Making

#### 3.1.1. Ambivalent Attitudes towards Retirement

The interviewees reflected on their transition to retirement and held both positive and negative attitudes towards retiring and the retiree role. The prospect of ending the working life was perceived as pleasant, as they would be freed from the obligations of work and have control of how to use their time; however, simultaneously, they were apprehensive that ending an important facet of their lives would feel strange, and they feared to miss having a job that added meaning to their daily lives and to become restless and empty. Conversely, those who considered themselves being further away from retirement had avoided thinking about it.

*First of all, it feels very unreal, ahem, I’ve not really taken it in yet. *(…)* No, but it brings, well it brings joy, that you will have time off, and be free and maybe do things that you yourself think are nice, to travel and such, but then again, it is a big part of your life that somehow comes to an end. *(…)* Yes *[laughs]* I think it will be weird *[the day she stops working]*. Then again, it might probably be nice too.* [Celine]

#### 3.1.2. Retirement Preferences

In general, the age of 65 was the preferred and most likely age at which the interviewees would start receiving their pensions. However, this was not necessarily perceived as the time to fully exit working life, and to continue working after 65 (but not full time) was in some of the nurses’ plans. Post-retirement work may take various forms, such as part-time employment, or hourly paid work, which was considered more flexible. Yngve expressed the flexibility of the Swedish pension system, which allows the employee to decide the time to begin receiving pension benefits within a certain age range and combine retirement with paid work.

*I will most likely retire when I turn 65, then I will stop working full time. And as a start, then I will use my pension benefits and work in some way, but I have not decided in what way. I have no idea whether I will continue to work on an hourly basis, or just work now and then, or continue to work part-time or something. But I will continue working in one way or another, I will.*

#### 3.1.3. The Transition to Retirement

Interviewees emphasized the importance of retirement planning and the voluntariness of the timing of the retirement. When asked about how he would feel the day he would stop working, Sten stated:

*Then I think I would have picked that day carefully; I would have decided for myself and be happy with it. (*…)* When I retire, I would have planned for it. *(…)* I know when to do it and I would have tied up all the loose ends ahem, and I would have nothing hanging over me and I won’t feel cast away. Because I think it’s important that you get to retire when you want to, not because your age stops you or that you become ill or something. *(…)* I think this difference is essential to feel that you’re done. It wasn’t working life that was done with me, but it was I who were done with working life. I think this is important.*

There was a clear preference for a gradual transition to retirement, as expressed by Daniella, who returned to work part time after regretting having fully retired.

*For me retirement isn’t… no, I’ve felt it has been quite difficult (*…)* ‘was it just this, eh? To make a cut?’ *(…)* I wanted to practice a bit* [the retirement life]. *(…) I’ve felt that it has been quite difficult to retire, the age, it’s not that fun to grow old *(…)*. And then I felt a bit like well *(…)*, this is probably good, to work part-time for six months and then I’ll cut* [completely]. *And here you can work on an hourly basis.*

Post-retirement work was seen as an intermediate state and a means to adapt smoothly to the retiree role.

*I think you need to have some place to go, and that’s the dilemma when you retire, that you have nowhere to go. Then perhaps you could work a bit as a nurse somewhere, on an hourly basis.* [Alice]

### 3.2. Self-Rated Health, Diagnoses, and Functional Diversity

#### 3.2.1. Being Healthy as a Condition to Continue Working

Despite not having the same energy as in the past, and needing longer to recover, interviewees were in relatively good health and did not feel that their age physically limited their work. Being healthy was seen as the most important requirement in considering working until age 65 and beyond.

*So, I don’t know how many years after my retirement I can work part-time, I don’t know, but if I’m healthy and feel that this is fun, then I can probably see myself working in the healthcare.* [Nina]

*I think it is most likely that I’ll continue working after 65, perhaps part-time, but I don’t know how my body will feel, but if I feel good and got the strength, then I think I’ll cut down to part-time.* [Petter]

#### 3.2.2. Being Healthy as a Condition to Enjoy Retirement

Besides being central for work ability, good health was also considered necessary to enjoy the retirement years. Fredrik, who planned to retire at 63, was worried about not having the health and time to do everything he would want to do after his retirement.

*But we also know that, especially we who work in this sector know that, it’s not a question of whether the diseases will come, it’s rather the question of when they will come. And then you also want to enjoy your retirement, I’ve worked well beyond 30 years. *(…)* It would be awful, really, if you were to come back as a patient instead. No, I wouldn’t want that. And not to get to experience all this that I want to do, go on all these fantastic cruises, there are a lot of retired people there, and that’s nice.*

### 3.3. Physical Work Environment

#### 3.3.1. Continuous Noise Causes Stress

The IC room was placed in a temporary building, and the room was very warm and considered small for the number of patients. The continuous noise from the equipment and the alarms was considered disturbing and stressful, and affected both the nurses and the patients.

*It is noisy and very warm. And then you must constantly be careful to avoid tripping on any cable hanging five centimeters above the floor *(…)* so that constantly, to be responsible for the patient’s life and at the same time be so *[aware of your surroundings]* *(…)* that you don’t hurt yourself or unplug a cable from any advanced equipment. *(…)* Eh, yes, I think it affects me and increases stress. *(…)* I don’t think anyone works well under pressure when it is noisy *(…)* and then you have to think about, it’s difficult for the staff but … what how about the patient? That is, patients who are lying down, who cannot turn over by themselves and staring at the ceiling or looking into a wall, and hear all these alarms, knowing that these alarms concern themselves, but don’t understand what they mean. *(…)* We know that we have patients who have nightmares about alarms after they recover from intensive care.* [Sten]

#### 3.3.2. A Physically Demanding Job

Working in an IC unit was considered to involve a comprehensive task list experienced as more physically demanding with aging.

*We care for* [patients], *we have to wash, care for, make their beds, turn, eh. Patients who are anesthetized, it’s very heavy, sometimes they weigh a lot too. They have to be moved in beds and sometimes, uh, we need to go for X-rays, tomographies *(…)*. I probably do the same amount* [as when I was younger], *but it’s a bit heavier, I cannot cope in the same way *(…)* it takes its toll on my body, I think. I think back* *when I was the same age as the younger nurses.* [Marie]

### 3.4. Mental Work Environment

#### 3.4.1. Being in Control despite the Unpredictable Context

Work was to a large extent perceived as unpredictable, as there were often unforeseeable events that interviewees needed to deal with when taking care of seriously ill patients. However, this apparent lack of control was not experienced as too stressful and was seen as an inherent quality of the job.

[I feel control over work] *to a fairly large extent, so you live in a chaotic world, because we live with people whose bodies have gone into chaos. We try, what we do is that we try to control this chaos, eh. Of course, it’s not possible, if we could, they wouldn’t have been in the intensive care unit *(…)*, but I strive for it* [control] *and I try to avoid thinking of the things that can go wrong, and if needed, to be prepared, materially and mentally. This is what an intensive care nurse quickly learns to do, to think about what might happen. *(…)* And once I have it in place, then I feel I have control, even if it *[something serious]* happens.* [Petter]

The IC unit stood out among others in the clear routines and guidelines, and this was felt as providing some sense of control when dealing with emergencies.

*I like working here, that’s why I came back too. *(…)* I feel that in this unit, things are in order, *(…)* there is a lot of routinesthat need to be followed. *(…)* I was away a few years and came back, I have been around and seen how it works in other places, I felt then that it worked much better when I was here, with the routines and everything like that.* [Helen]

*That is, when the environment becomes stressful, when we are pushed to our limits, then we need have a working communication system and stick to it. Avoid running, stay in the box, and know your function.* [Sten]

#### 3.4.2. Job Demands—The “Ethical Stress” and the “Nursing Dilemma”

Work was seen as mentally demanding. There was a heavy workload in which critical moments were felt as difficult to handle. In these situations, it caused what Marie called “ethical stress”.

*Sometimes the workload becomes so high that you don’t have the control that you need to fulfil patient safety. *(…)* It’s difficult, so difficult, it’s the most difficult thing about this, it is the most difficult part of the job, the ethical stress, not being able to meet adequate needs in an adequate way.*

Petter, in turn, talked about the “nursing dilemma”, that is the need to make priorities in the care that is to be provided and to delegate some tasks to nursing assistants.

*There is a constant frustration in not having time for the basic nursing, I don’t have time to carry out the core of the nursing profession, you have to hand it over to assistant nurses, and this is far from good. *(…)* We have far too much, we have more to do than we squeeze in on a working day, that is the nurse’s dilemma.*

Work was sometimes experienced as emotionally demanding. Interviewees also mentioned the conflicting demands and described the stresses of such demands.

*Because, you can stand there and mix a very important infusion that will soon run out in a seriously ill patient, and then something else happens to the other* [patient], *which needs to be taken care of immediately. It gets extremely stressful, ahem, if you’re lucky, the colleague in the room next door may help you, but sometimes you can end up in almost impossible situations*. [Marie]

Furthermore, nurses felt they were responsible for their patients’ lives. In this, having to handle the relatives of the patients, while being perceived as an important task, still involved an additional strain.

*‘Too many irons in the fire’ and you work with human life, yes. So, my evaluations affect patient health, or what happens to the patient, we handle very advanced drugs, and very advanced technological equipment. If we do anything wrong, the result can be bad, so it is always important to focus.* [Ida]

Ultimately, the demands of the job can be a motive for stopping to work in IC after the age of 65. Marie was especially critical of the recent changes in the Swedish pension system not taking into consideration the type of occupation, as some occupations including those in healthcare, seem more demanding than others.

*But I really think they should differentiate that because ahem, assistant nurses for example who work hard, it’s not okay, ahem… you sit in the office, I don’t say that you don’t work, but it doesn’t add wear and tear to your body. *(…)* Like lifting a lot, because they are the ones who do all the heavy lifting.*

She added that in the nursing occupation, the lack of energy and work ability may have different and more serious consequences than other occupations.

*When we don’t really keep up with everything as we should, then it’s people who are affected, and it’s a huge difference between having to wait a long time for pain relief or having to wait a long time for a newly bought car to be delivered.*

#### 3.4.3. Job Demands Also Perceived as Positive Challenges

Notwithstanding experiences of stress at work, interviewees generally considered this to be manageable to a certain extent, as expressed by Ida, who felt that she could rapidly recover from stress.

*Eh, yes it* [stress] *is about not always having time to do what you have to do. It’s an issue of having too many ill patients, it is too demanding to deal with. It can be stressful but it is not something that affects me long-term, only the moment when it happens.*

Similarly, Marie, even if she perceived her job to involve various types of demands, felt she could cope well with them, *“Ehm, it’s going well. I’m tired in my body after a day, but I’m fine. I do exercise, I’m careful to keep myself physically active in my free time too!”*.

Furthermore, job demands were also positively perceived as providing the nurses with some challenges, which were appreciated and a job characteristic nurses would miss when fully retiring.

*This is a bit of an intellectual challenge, so it’s not just a job that you go to and* [plodding along] *like this. You have to be alert and attentive all the time. *(…)* above all* [what I would miss the most are] *these challenges at work and solving the problems*. [Helen]

### 3.5. Work Schedule, Work Pace, and Recovery Time

#### 3.5.1. Irregular Working Time May Be a Hinder for a Sustainable and Healthy Working Life

An IC unit functions 24/7 and requires healthcare staff to work days, evenings, nights, and weekends. In this particular unit, all nurses were required to work in two shifts and some weekend days every month. However, there was some flexibility and, whenever possible, the work time would be scheduled according to the employees’ preferences, which was very appreciated by the nurses, even if it was not always possible.

*Maybe that you would work less during the weekends* [would facilitate extended working lives]. *Above all, one thing, and it works here, is that you don’t have to work three shifts, but there can be a choice. There are many units where you have to work three shifts, which many find very tiring. And not having to work nights when you’re older, *(…)* then you just work days and evenings. But this, well it already works here, for everyone*. [Helen]

Cecilia felt that shift work was particularly demanding, and was happy to be able to work only the day shift two weeks a month, which allowed for some stability.

*It’s difficult to have irregular working hours, I think it wears more on the body ahem.* [Working more days and having more weekends off] *means a lot, I think, I have longed for it a bit, to be able to do this, and it is great *(…)* it makes wonders for the routine, I sleep much better.*

Working nights was considered demanding and a health risk, as mentioned by Helena, who no longer did night shifts.

*Working nights, I felt that, no, it wasn’t for me. My body and mind simply could not cope; it didn’t work at all. I couldn’t sleep, neither during the days nor nights, I slept very badly, and now I have, I just feel like this, that now* [after stopping working night shifts], *I go home and just go to bed, and then I fall asleep.*

However, the nurses did see advantages of working nights and weekends, as it provided an extra income, reduced the number of weekly work hours, and provided some free days during the week. Moreover, it allowed some variation and work in a calmer environment.

*I* [like] *working weekends, I like having weekdays off. *(…)* There are less people* [at the unit] *and the fewer you are the less messy it becomes, it’s a bit quitter in that way.* [Nina]

In the long term, irregular working hours were nevertheless seen as a potential hindrance for extended working lives.

*I don’t know how I will feel in ten years, but it may be the working hours that are tiring in the long run. *(…)* You get more tired in the future *(…)*, so it’s quite tiring to work late evenings and get up early in the morning, and work night shifts maybe, and you may not have the energy.* [Ida]

#### 3.5.2. Recovery Strategies

To work with seriously ill patients did not allow taking breaks at regular times of the day, as the breaks were dependent on the tasks that needed to be performed, and it was not always possible to predict the duration of such tasks. The nurses had no possibilities to take longer breaks as they were needed at the unit: *there is a person there, a seriously ill person, and I need to be there*. [Marie]

Sometimes, the nurses experienced difficulties recovering from critical events at work: *Sometimes, sometimes, it depends on how tough [it is] but, it can take weeks to handle events at work [such as] patients who have had bad experiences. (…) I’m tired for days.* [Petter]

Furthermore, nurses felt a greater need for recovery as they were aging.

*I have a greater need to rest and recover, I do. *(…)* Sleeping, that’s what I need to do. I feel that, I didn’t need that before, then I could stay up much longer, but over the years I feel more tired.* [Alice]

To decrease one’s working time was seen as an important strategy to increase the time for recovery and to protect long-term health: *It takes many, many hours before I unwind. I’m tired the next day. I work part-time* (…) *To have a little more recovery*. [Marie]

However, this impacted on income and future pensions.

*I don’t work full-time* [works 90%], *I’m absolutely sure that I won’t work full- time [until retiring], very few nurses above 30 years of age want to work full-time. *(…)** [I work] *90* [%], *which is too much. *(…)* I would like to cut down my working hours. But then, well, it affects my income and pension, so this is what holds me back.* [Petter]

While the nurses welcomed the possibility to work part time, there was concern that this would lead to a loss of competence.

*And at the same time, I think that *(…)* you shouldn’t work too few hours. Not in healthcare, if you are to continue working *(…)* because then you might not be able to keep up, then it will be too burdensome. So, there may be a limit to how few hours you can work if you are to stay.* [Lotte]

To perform other tasks outside the unit was also a strategy to recover from the particularly demanding job as an IC nurse and to facilitate a longer working life in the nursing profession.

*Getting a break, if I worked here full-time maybe I would not be as happy, maybe I would consider it to be more tiring. But I get a break by doing two different things and that’s satisfying, yes.* [Ida]

#### 3.5.3. Post-Retirement Work as a Way to Work in a Flexible Schedule

Post-retirement work was seen as a way to gain control over one’s work time and to have a more flexible schedule. This freedom, along with the possibility to remove undesired tasks and responsibilities, seemed very attractive to nurses when they thought about their late career options.

*Because I’m really tired of working weekends, I’m really, really tired of it, so it would be nice* [to be able to decide when to work or not]. **(…)* When working on an hourly basis, you can have a lot of influence* [regarding decisions when to work]. **(…)* Now I cannot influence this that much* [the working hours]. *We put in, we have a wish list. So, we can influence it to a certain extent, but, when working on an hourly basis, you can influence this a lot*. [Daniella]

### 3.6. Personal Finances

#### 3.6.1. Financial Considerations

Although the nurses thought that they would have a relatively comfortable economic situation when retiring, one of the attractive factors in working until the age 65 and beyond was to guarantee a higher pension-related income after retirement.

*I like money* (laughs), *to get a little more, increase the pay desk actually *(…)*. Not because I have a huge need, I’m fine. I can manage, absolutely. *(…)* But I still think that this feeling of getting some money and feeling that you have done something would be good, would be good, at least in the beginning* [of the retirement life]. [Alice]

### 3.7. Personal Social Environment and Private Life

#### 3.7.1. Attitudes towards Leisure

Notwithstanding the diversity regarding the relative importance that work and leisure had in their lives, the nurses’ overall experience was that work did not prevent them from enjoying their time off nor from engaging in family and leisure activities, and they felt they had a relatively good work/personal life balance. To decrease the working time was nevertheless seen as attractive and a possibility for more leisure time, if they were economically independent. The interviewees reflected on their aging and were increasingly aware of the fact that life is limited in time. Their future time perspectives, and the importance of having a fulfilling private life in their remaining time, while still keeping up their health, played a role in the attitudes towards leisure.

*And, I have a limited time left, statistically I’ll only have about 23 years left to live, and out of those 23 years, maybe I have 15, what do I know, with a body that works, when I still think it’s nice to do things, *(…)* so I don’t have so many years. *(…)* Then I need to use the years I have left and spend my time on nice things. *(…)* Most* [of the dying people] *regretted what they didn’t do. Knowing this, then I think that I may well try to do nice things *(…)* in the years I have left.* [Yngve]

Despite this awareness of a limited life time to engage in meaningful activities, Yngve acknowledged that work continued to play an important role, in having a relevant social function, as was the case, to some extent, for the other nurses too.

*I think I value my work more today. I took it more for granted earlier eh, also, *(…)* it means more. But it can also be me being in a different social situation than I was before. *(…)* Yes, so I have fewer social contacts, that’s why I agree that I have to create a social life outside work and I do that gradually as well.*

#### 3.7.2. Family Situation Plays a Role in Work Centrality and Retirement Plans

The family context played a role when reflecting on the importance of work, the timing of retirement, and engaging or not in post-retirement work.

*It* [work] *has a big place in my life, I think what I do is fun, and since I don’t have a family of my own, then work takes up more space. *(…)* If I had my own family and grandchildren and all this, then I don’t know what it would have been like. But since I don’t have it, work adds a dimension, yes, it gets a bigger space.* [Marie]

There was a wish to coordinate retirement plans with the partner, as recognized by Celine:

*Yes, my husband of course* [will affect my retirement decision]. *He’s six years younger than me, so we have talked a lot about, how we should handle it, because it is perhaps not so fun to go home and wait for someone for six years, it has to be some kind of compromise*. [Laughs] *Yes, but it makes me think that maybe I should work ahem, a bit on an hourly basis, so, I’ve started to think, or we’ve discussed it a bit, and that he might also do that, so that we can get more time off together.*

The existence of (grand)children or parents to care for was also referred to as an important factor to take into account in the retirement decisions.

*I want *(…)* to try to spend more time with grandchildren, so perhaps you can pick them up from preschool, *(…)* if you retire, you can then set aside one day a week when you do these things, well that’s it. *(…)* My parents are still alive ahem, yes, you talk about it* [retirement] *a bit, and so. *(…)* Yes, my parents think I should leave earlier* [laughs]. [Celine]

### 3.8. Social Work Environment, Discrimination, Leadership Style, and Age Management

#### 3.8.1. Organization-Based Self-Esteem

The nurses felt very respected and appreciated, both by their colleagues and managers. Their long experience and knowledge were recognized, and they thought they had a valuable and important role at the unit.

*I’m a very experienced nurse ahem, and I also get recognition for that, both from colleagues, but also from managers. *(…)* Colleagues, both nurses and assistant nurses, come and ask, ‘What do you think?’ ‘Would you, can you have a look?’ ‘What would you do in this situation?’* [Managers say] *that I’m capable, that I’m being helpful, that I don’t snap back at them and that I *(…)* ‘you do a good, a very good job’ and that I’m one of the most competent, they say. *(…)* And that, I have that with me, and then I feel that I can be happy that the colleagues I work with encourage me and say that we have… we work well together.* [Lotte]

#### 3.8.2. The Importance of a Supportive Working Team

The social work environment was perceived as very supportive, which was considered to play an important role in balancing the impact of job demands as well as for the satisfaction the nurses mentioned having at work, which in turn contributed to the motivation to continue working. Colleagues were a strong asset and a vital resource, particularly when handling critical events involving patients, which were common at the unit.

*I always feel secure when I work together with other people, *(…)* we always work in a team, we do it together, and it works incredibly well at this hospital, I think, this unit where I work, that we work together. So, I’m confident that if it’s not me, there’s always someone else who can do it. And we help each other, and then it works well.* [Yngve]

#### 3.8.3. A Supportive Leadership Is Central for Job Retention

The nurses were very satisfied with the leadership at the unit, which they described as having the ability to motivate the group.

*I’ve never been at a workplace where so many are so positive regarding their manager as here, it’s fascinating, because I’ve worked for a long time and been at many different places, so he is very good, he is here and he sees us and he is there for us. *(…)* I feel this all the time. *(…)* And I also know other hospitals have concerns regarding the salaries and work time models, and stuff like that, we don’t have to think about it that much. We know that this will be sorted, and so do they, so they* [managers] *do that. So, I’m very happy*. [Yngve]

Managers, both those at the front line and at a higher hierarchical level in the unit, were perceived as being present, accessible, open, fair, and close to the employees. To give honest and timely feedback was also mentioned as an important quality that they recognized in their leaders.

*I think that it is* [a fairly open climate], *they also deal with things. They* [the managers] *work, they are a bit different, but everyone tries to provide feedback *(…)** [a good manager] *should be able to see me. *(…)* She should be able to provide feedback.* [Lotte]

The feeling of having a supportive leadership played a central role in job satisfaction and their willingness to stay at the unit and continue working.

*I think he is understanding and he, if you have something, you can go and talk to him and he will listen, and he takes his time and so on, he does. *(…)* He is extremely popular among the employees in the unit. *(…)* We have many employees who have worked here for many years, and I still think that part of it, why you stay, is because you know you can reach your manager and meet them.* [Alice]

#### 3.8.4. Age-Friendly Work Environment

The working team was age diverse and age neutral, and the nurses did not feel that they were discriminated against due to their age. They welcomed the opportunity to use their accumulated knowledge and experience to help their younger colleagues and simultaneously felt that they could learn from the younger ones.

*I think it’s great* [to collaborate with younger employees], *ahem that you feel much younger and then it’s they who know about the latest findings. It’s been a number of years since I went to school. *(…)* I learn from them and they learn from me, so that, yes, I think it’s a lot of fun. *(…)* They know that you’ve been through a lot more and have a lot to teach. *(…)* They ask, ask questions, come and ask for help, want to know ‘that I’m doing the right thing’, that is it.* [Nina]

#### 3.8.5. Age Management Practices That May Promote a More Sustainable Working Life

The nurses did not find many possibilities to adapt some of their responsibilities and working tasks to their age, potential changes in work ability and interests, and thought that work would become more manageable in a long-term perspective if such adaptations were possible.

*But it’s still like that you have the same expectations on yourself, as when you started working as a 23-year-old, as when you leave as a 65-year-old with 45 years of experience. You should do as much on your first day as you should do on your last day, that’s absurd.* [Petter]

As Marie noted, increasing the staff would allow for a decrease in the workload and facilitate extended working lives: *Increased staffing. *(…)* That you didn’t have to stress around so much, and hurry through the whole workday. We often drive at full speed the entire work shift, and it is not healthy*.

Nina, in turn, welcomed the possibility for lateral job movements.

*If you could combine not only working on the floor, but maybe get some administrative *(…)* administrative tasks or something else on the side. *(…)* That is, if you get older you won’t have as much strength, it will be heavy to run around here, and stand, lift. So that, making use of the knowledge that the older nurses have.*

The nurses said that they sometimes assumed a mentoring role. However, this was perceived as rather informal and not organized. To formalize such a role and promote the systematic transfer and knowledge exchange with the younger colleagues was seen as a practice that would benefit the unit and facilitate extended working lives.

*That you are some kind of senior nurse and walk around and support those who don’t ahem, these newcomers who have just arrived, and be a support, but then, maybe not have so many patients of your own, but to work more, like a mentor maybe. And support the* [younger nurses], *to help them out in these situations that turn out to be more demanding, and in the daily, well the regular work as well.* [Helen]

To retire and engage in post-retirement work was considered as an attractive strategy to skip some of the responsibilities at work while continuing to work with more suitable and interesting tasks within nursing: *As it is now, it’s the case that when you turn 65, then you get rid of these other tasks, as you no longer have a permanent position*. [Lotte]

*There is a responsibility that you let go of. A responsibility in a broader perspective, which I think I should have, and I have, for the workplace. It doesn’t continue, I’ll then* [after retirement] *come in and solve specific issues, and then I don’t have to solve the problems with the storing of the medication, or stand in the sorting room, and things like that.* [Marie]

### 3.9. Motivation, Satisfaction, and Stimulation in the Execution of Work Tasks

#### 3.9.1. A Rewarding and Meaningful Job

When asked the “lottery question”, the nurses reflected on motives for work. While the income was a strong motive, they would continue to work to some extent even if they would receive a large amount of money.

*I would probably like to work less, yes, I would probably like to do that, but I wouldn’t want to stop working, no. *(…)* Because I think it’s fun to work, it’s a satisfaction, it’s as I say, all the people you meet enrich your life.* [Ida]

*I don’t think* [money] *is a driver of whether I should work or not, I think I would like to continue working in any case, even if I would end up in a financial situation where I wouldn’t have to work another day in my life, but I don’t work for the money. I actually work for the feeling of being in a context where I’ve something to contribute and where I feel good about it, so that I actually don’t think that… it’s not for the sake of my own profit that I do things*. [Sten]

The interviewees thought that their work as nurses in an IC unit, where their daily focus was saving the lives of patients with extremely delicate health conditions, was very rewarding and meaningful: *It* [the job] *gives incredibly much back, a job in healthcare, it gives a lot back, at the human and social levels, so that is perhaps what I value most, yes* [in having a job]. [Ida]

The meaningfulness of work was considered as a strong motive to continue working beyond the age of 65.

*I don’t think I could quit the job* [if I won the lottery], *I don’t think so *(…)* it’s the incredible need for acknowledgment, I think. But to feel that I mean something *(…)* to me is an important part. *(…)* Because I know what drives me somewhere and what I think is important. *(…)* I’m a bit scared when I quit. Just this feeling of being needed and being able to really give things and get the acknowledgment. *(…)* Because a day, it ends, when I don’t work then. Then I just hope I’ve found myself in that ‘now it’s enough, now I have other things I want to do’.* [Yngve]

#### 3.9.2. Variety at Work

The nurses very much appreciated the variation they found in their work. They enjoyed the unpredictability and working in a place “where things happen”. Moreover, feeling that their job was interesting and stimulating influenced their willingness to continue working beyond retirement.

*It* [willingness to work beyond 65] *is probably partly because I think it’s fun, partly because I think I can do it, and it’s stimulating, intellectually stimulating, socially stimulating, and financially stimulating.* [Petter]

#### 3.9.3. Job Satisfaction

The interviewees mentioned being very satisfied with their jobs as IC nurses and with the organization, and reflected on this in different ways during the interviews. Job satisfaction was central for staff retention, and for the openness to extended working lives.

*As long as it feels like this and I’ve a good workplace to go to, I’ll *continue** [to work full time until retirement]. **(…)* Because I enjoy my job. *(…)* The organization works well *(…)* it is good on so many levels. *(…)* I don’t think I would have this elsewhere.* [Yngve]

### 3.10. Competence, Use of Skills, Knowledge, and Opportunities for Development at Work

#### 3.10.1. Opportunities for Use of Accumulated Knowledge and Competences

The nurses very much valued the opportunity to use the specialized knowledge and range of competencies that they developed throughout their academic and working life.

*Mm, I think *(…)* that my knowledge is used in a good way. *(…)** [what you appreciate most about having a job] *is, ahem, it’s simple, it’s to be able to work with what I’ve been trained for, that’s right*. [Celine]

#### 3.10.2. Occupational Self-Efficacy and the Positive Side of Being an Older Worker—“the Clinical Eye” and “the Tacit Knowledge”

The nurses were very confident of being competent to perform their job well, as well as when dealing with critical events: *I can see, immediately take things in* [I have to do this, I have to do that]. *It’s a clinical eye, yes, it’s a clinical eye*. [Sten]

The “clinical eye”, resulting from the experience accumulated during the years, provided nurses some calm, and to some extent buffered the impact of both job demands and lack of control, which were seen as inherent characteristics of the IC job.

*Ahem, it’s because I’ve been here so long, and been through so much, I think. Then it feels like I, I can handle most situations, then I perhaps think that things can be more or less difficult, but I can usually handle it.* [Celine]

Fredrik referred to it as “tacit knowledge”, which is the way his experience is being expressed at work.

*This tacit knowledge. So, you’ve been involved in and through a number of things, then you get a feeling for the situation, and you also get a feeling for the course of events. And it’s very, very difficult sometimes to teach, but it’s something that you learn when you’ve experienced it a number of times and I think it, it’s actually really invaluable. *(…)* You have, you develop some kind of emotional antennae, “what are things going to be like for this patient?” Although you don’t see any signals yet, older nurses have already their heads wrapped around it, yes.*

The long work experience also helped to recover emotionally from tragic work-related events.

*But before, I would probably turn in my bed for quite a long time* [after a tragic event at work], *but I have no problem with this anymore, I can go home and go to bed and sleep. *(…)* It* [what helps a faster recovery] *is the experience, absolutely, and then the more you see and the more you come across, so yes, you gain a lot of knowledge from different experiences, you use it too, I’d say.* [Helen]

#### 3.10.3. Importance of Keeping Skills Up-to-Date

The organization provided opportunities for training and development, and overall, the interviewees considered themselves to have had their skills updated: *Good* [opportunities for skills development], *I think.* (…) *A lot of courses* (…) *we have a continuous training at work, we have a lot*. [Petter]

This was highly valued and recognized by the nurses, who considered it important to follow the development in their area of expertise, despite not feeling any need for extensive training, given their seniority and being near retirement.

*I want to develop my competencies very much, I want that *(…)* because you can always learn something new, and there will be new findings as I said before, so all who work at the unit need to take part, I’d say.* [Celine]

#### 3.10.4. Internal and External Employability

The interviewees mentioned that as senior, competent, and experienced nurses, they had good opportunities to continue working after retirement, at the same, or at some other, hospital.

*Oops, *the** [current job market in healthcare] *is huge. There are many job opportunities, both in Sweden and Norway. *(…)* There is no age discrimination, instead they appreciate that you are experienced and stuff like that, I’m still very much in demand on the labour market*. [Yngve]

The high perceived internal and external employability seemed to have a strong impact on the motivation to continue working.

*Yes, but we have several of those who retired recently, they are back, working. There’s no difference. So, most who retire return *(…)* and work on an hourly basis. *(…)* I think I have the prerequisites* [to continue working after retirement]. *I have a job that I’m happy with, *(…)* I have endless opportunities to continue working with things that I know. And then, I mean, I can do it until that day I that say ‘no, now I’m happy, now this is enough’.* [Sten]

## 4. Discussion

This interview study investigated older IC nurses’ experiences of working life and their reflections about the late career and retirement. The interviewees provided rich accounts of their experiences, and the interpretative phenomenological analysis resulted in ten superordinate themes, with the first centering on the nurses’ retirement decision making and nine themes reflecting the nine determinant areas proposed by the SwAge model [59] involved in individuals’ decisions on whether to extend their working lives or to retire. The discussion of the results will follow the thematic structure identified in the study.

### 4.1. Retirement Decision Making

Retirement is a major life transition [60] that may have a deep impact on individual lifestyles and social networks. About 10–25 percent of older workers have difficulties in adjusting to retirement [61]. Thus, retirement is a transition that can generate an ambivalence, as the individual, in the moment of choosing whether or not to retire, often considers both positive and negative outcomes of work and retirement [62]. Indeed, the nurses participating in this study expressed ambivalent attitudes towards the retirement transition and the role as retiree; this included both positive and negative valence. Moreover, some of their retirement plans included continuing working after the age of 65, which reflects the flexibility of the Swedish pension system and its facilitation of a steady transition to full retirement and extended working life. Importantly, retirement was perceived as a new career stage [24,63], with post-retirement work being considered an opportunity to engage in new roles in a more attractive employment form, while simultaneously allowing for a gradual disengagement of the working role and adaptation to life as a retiree. Post-retirement work may help individuals to maintain their identity of a work role [20], to avoid an abrupt discontinuity of their life structure after retirement [64,65,66], and to achieve a balance between the gradual distancing from work and continuity in life [67].

The importance of planning for retirement and the voluntariness of retirement were mentioned by the nurses. Indeed, Shultz and colleagues [68] found that the extent to which a retirement decision was perceived as voluntary or not impacted on retirement wellbeing. Importantly, individuals who retire due to different factors forcing them out of the workforce (so-called “push factors”, such as poor health or skills obsolescence), report poorer retirement wellbeing [68,69]. Although retirement planning and preparation is considered beneficial to the quality of adjusting to retirement [70,71], it is not frequent among older nurses [40,72].

### 4.2. Self-Rated Health, Diagnoses, and Functional Diversity

The nurses reported having relatively good health, without any significant physical ailments. In an occupation frequently associated with back pain, musculoskeletal disorders, psychological distress [48], and high turnover rates [38], they can be considered “survivors”. Consistent with the literature, the nurses in this study considered health as a major requirement for continuing to work. While good health has been associated with preferences for late retirement [73,74], poor health is indeed one of the strongest predictors of early retirement [20,23]. De Wind et al. [75] identified four pathways through which poor health leads to retirement: (1) the employee’s total incapacity to work due to health problems; (2) the employee being pushed out from work by the employer due to health problems; (3) the employee’s perceived decline in the (future) work ability caused by poor health; and (4) the employee’s fear of further health declines. The latter two pathways have also been found by Pond et al. [76] (p. 527) and were named “the health protection pathway”. Moreover, the current study findings showed that health was considered a pre-condition for enjoying the retirement years as well, and the nurses expressed that they wanted to retire while still having a good health, which aligns with findings reported by De Wind et al. [75] and in Pond et al. [76], where this pathway was referred to as the “maximization of life” exit pathway (p. 527).

### 4.3. Physical Work Environment

The physical work environment includes features such as noise, air quality, cleanness of the space, total area of the premises, temperature, and lighting [77,78]. The quality of the physical healthcare environment influences the quality of healthcare provision [78] and older nurses’ retirement timing [16]. The nurses in the present study complained with regard to their physical work environment, in particular the small rooms, with its implications for their and patients’ safety, and the constant noises coming from the life support equipment. The noise level is a particularly relevant work stressor with a negative impact for the nurses’ performance [77]. As older nurses are more prone to physical injuries, a good physical work environment that is well-equipped and has a reasonable sound level is an important factor that adds to their job satisfaction to a larger extent than it does for younger nurses [45]. Importantly, the nurses perceived nursing work as more physically demanding as the nurses became older. Research has found that working in more physically challenging environments is perceived by older nurses as tiring and a challenge to their continuity in the nursing profession [79]. Handling patients, including the repeated bending, lifting, and twisting may result in musculoskeletal injuries. Such injuries may accumulate over time and add to other physical problems that can occur with aging and thus make older nurses more vulnerable to the occupational risks that are commonly found in healthcare [13,15,80]. This means that organizations need to consider these issues, and make efforts to promote the use of ergonomic aids and design functional, comfortable, and safe workplaces, which provide a healthy and sustainable working life for all ages [15,59].

### 4.4. Mental Work Environment

The lack of control experienced by the nurses was seen as an inherent characteristic of their work with severely ill patients at the IC unit. However, the nurses considered the clear guidelines and routines at the unit as adding some control, even in the case of unforeseen events. The nurses experienced stress and pressure that came from a heavy workload, which led to time pressure and feelings of insufficiency, guilt, and frustration, and fears of risking the quality of patient care. Overall, they felt being capable to meet the demands of the job, but excessive job demands would still be a reason for not continuing IC work after retirement. Yet, the job demands were to some extent perceived as positive challenges and motivating job characteristics that the nurses would miss when leaving the profession. This follows Furunes and colleagues [73] finding that older workers appreciated high job demands, due to this making their work more interesting and challenging. Furthermore, a decrease in such demands was a reason to retire and not continue working. Importantly, these findings align with distinguishing between “job hindrances”, that is, the demands that elicit negative emotions, and “job challenges”, which refer to demands that, despite depleting energy, have stimulating characteristics [81]. Similarly, LePine et al. [82] in a two-dimensional model of stressors and performance, distinguished between “bad stress” and “good stress”, with the first resulting from hindrance stressors, including the inadequacy of resources, role ambiguity, interpersonal conflicts, and the latter resulting from challenge stressors (such as job demands and workloads) that may be associated with high performance. Providing the access to resources and practices to manage stress and reduce the levels of strain (such as support, time for social and physical activities, and training to prioritize tasks) may protect against any costs of challenge stressors for the long-term health of nurses [82].

### 4.5. Work Schedule, Work Pace, and Recovery Time

The opportunity to control work time is considered important to older nurses’ job satisfaction [45]. The nurses taking part in this study reported that the unit had a two-shift working schedule. However, older nurses were not required to work night shifts, which has been identified as a type of work schedule that is associated with nurses’ intentions to leave the organization [32]. Moreover, managers tried to consider the nurses’ preferences for shift schedules, something that was much appreciated by the nurses. Indeed, the results of a large longitudinal European study [83] has shown that fulfilling nurses’ wishes regarding shift work patterns may sustain nurses’ work ability and health, which in turn facilitates extended working lives. Moreover, Leineweber et al. [35] found that satisfaction with schedule flexibility was associated with a lower intention to leave the nursing profession or the organization.

Irregular working hours or rotating shifts are typical stressors of nursing work that become more difficult to handle with aging [13,48]. In the present study, this emerged as an occupational hazard that, in the long term, would potentially hinder the nurses’ ability to extend their working lives. Yet, this can be balanced by flexible schedules and freedom in choosing when and how to work. These were characteristics of post-retirement work that the nurses considered as very attractive—a finding which follows previous studies of older nurses [42,50,84,85]. The majority of the nurses that were interviewed worked part time. This was a strategy to increase recovery time. Importantly, recovery was perceived as key for health, wellbeing, and the ability to perform at work, and consequently, for remaining longer in the nursing profession. Another recovery strategy was to distance oneself from the IC work, and to carry out other types of activities considered less emotionally laden outside the unit.

### 4.6. Personal Finances

Financial resources available for retirement is one of the most frequently recurring factors in older employees’ conceptualizations of extended working lives [20,23]. However, the role of financial factors for extended working lives is not straightforward [86,87]. While some studies show that higher pay and pension incomes are negatively associated with the likelihood of post-retirement work [88], other studies find that wealth does not predict the decision to continue working after retirement [89]. Some findings suggest a U-shaped relationship, according to which post-retirement work participation is higher among individuals with lower and higher retirement incomes, as compared with those in the middle [90]. The nurses interviewed in the present study considered finances to play a role in their retirement plans. However, this was not the core motivational driver for continuing to work, which is something that has been found in other qualitative studies with more heterogeneous samples [47,91,92]. The nurses thought they would continue working to some extent even if they would win a large amount of money in the lottery. Rather than financial needs in its strictest sense, the main finances-related motives for working until the age 65 and beyond seemed to involve a guarantee of a higher pension income, to maintain a certain living standard, and to afford some extras, such as traveling.

### 4.7. Personal Social Environment and Private Life

The nurses mentioned that being engaged in several activities during their leisure time, including having a relatively good work/private life balance, was important for their wellbeing. However, there was an increasing awareness of the fact that a human’s lifetime is limited, which created an urgency to make the best of one’s remaining time. This finding can be related to the socioemotional selectivity theory [93], which argues that perceptions of the amount of time left in life impact profoundly on individual motivation and priorities. Importantly, when perceiving that time is limited, individuals seem to become more motivated to prioritize their emotional satisfaction, and focus on deepening their relationships, and on enjoying life.

The mentioning of work having a continued and important role in providing opportunities for socialization, particularly for those not living with a partner, has been found in other studies of older nurses [27,45,49,50]. For instance, continuing to work due to social reasons seems particularly common among older women [94]. Yet, the importance of having time for family life and sharing leisure activities has been reported in previous research as well [16,43,46,48]. Specifically, nurses with a partner expressed a wish to coordinate their retirement and the potential engagement in post-retirement work with those of their partner. These findings suggest that healthcare organizations that facilitate work–life balance, through flexible work arrangements, may delay full retirement of older nurses who may wish to retire at the same time as their partners, who may be older and entitled to retire earlier.

### 4.8. Social Work Environment, Discrimination, Leadership Style, and Age Management

The extent to which employees perceive themselves as capable, meaningful, and valuable to the organization employing them has been referred to as “organization-based self-esteem” (OBSE) [95]. OBSE has been linked to employee motivation, job satisfaction, organizational commitment, performance, and mentoring behaviors, as well as to lower levels of both turnover intention and actual turnover [95]. The nurses participating in the present study mentioned being respected and appreciated both by their colleagues and managers at the IC unit. Importantly, both colleagues and managers recognized the value of the expertise and the quality of the care that the nurses provided. Similar to previous studies of older nurses [28,45,48,84,85], the esteem and appreciation reported by the IC nurses seemed to play an important role for their satisfaction, but also for their commitment and sense of belonging to the workplace. This has been underscored as important to consider for organizations, for instance by implementing a “culture of appreciation” to attract “silver workers”, or active retirees, in order to recognize the older employees’ accumulated experience and expertise [96] (p. 152).

According to the job demands–resources (JD-R) model [97,98], social support is a job resource that promotes work engagement, and buffers the impact of high demands, particularly in highly demanding jobs [97,99], such as IC nursing. Here, the nurses mentioned getting much support from their colleagues, which they considered an important resource that helped to counterbalance the negative impact of work stressors. Moreover, the support seemed to increased their job satisfaction, commitment to work, and health. This aligns with a systematic review [100], which reports linkages between social support and lower risks for burnout among emergency care nurses.

The leadership was also perceived as very supportive, in contributing to a good work environment, which in turn seemed to have a positive impact on the nurses’ job satisfaction as well as on their willingness to stay with the organization. This finding follows those of a systematic review of the effects of leadership styles for nurses [101]. Specifically, leaders who make use of their emotional skills and are empathetic and responsive to work concerns and emotional needs of the staff, and promote the best use of staff competencies and expertise, seem to empower nurses while also adding to their job satisfaction, satisfaction with the leader, productivity and effectiveness, and organizational commitment, as well as to reduced retention. Importantly, such leaders, in making use of their skills, contribute to a variety of positive outcomes for the nurses, for the work environment, and the organization [101].

The nurses who were interviewed did not feel being discriminated against by their age, and were very committed in passing on their knowledge and expertise to younger nurses, thus showing high levels of generativity [102]. In a recent meta-analysis [103], this has been found to have several positive consequences, including job satisfaction, work motivation, occupational self-efficacy and a motivation to continue working. Here, the nurses mentioned that their mentoring of entry-level nurses was rather informal and not systematic. Thus, the interviewees suggested that creating formal mentoring programs including senior and more experienced nursing staff would be a practice that would encourage employees to extend their working life in the organization, something that has been suggested in the literature as well [13,17,42]. Moreover, the nurses thought that their work would be more sustainable in the long term if they would have the possibility to reduce their workload and reassign some responsibilities and working tasks as a way to address their interests and potential limitations related to aging. This means that they suggested the organization to offer older workers opportunities to engage in job crafting behaviors in order to promote person–job fit and foster successful aging at work [104], to extend their working lives.

### 4.9. Motivation, Satisfaction, and Stimulation in the Execution of Work Tasks

The nurses mentioned being very committed to their work and satisfied with their jobs and considered this to be important for prolonging their working lives—a finding that has been reported in previous studies as well [16,45]. For instance, previous research has found a U-shaped relationship between nurses’ age, seniority, and job satisfaction with higher levels of job satisfaction at the entry level and after the age of 50 [105]. For the older nurses, this may be explained by an increased expertise, better working schedules, and salaries, and better work–family balance. However, for older nurses, the increasing job satisfaction may reflect a selection effect, that is, the less satisfied and the dissatisfied nurses may have left the job. Such a selection may also apply to the present study findings.

The IC nurses felt intrinsically motivated to work, that is, were driven by the work content and the inner joy of the work itself cf. [106]. As has been suggested [80,107], the intrinsic rewards of healthcare may promote work ability as well as work engagement. In particular, work engagement may counteract burnout [107], which is common in healthcare occupations. Here, the nurses described their nursing work at the IC unit as varied, interesting, and stimulating, as well as very rewarding and meaningful. Importantly, this was a strong motive for continuing to work beyond the age of 65. This finding is in line with previous research [74,108], showing that individuals who extend their work careers typically feel important to other people and that they have meaningful tasks.

### 4.10. Knowledge, Skills, and Competence

In the present study, the opportunity to use skills and knowledge acquired over the years of work seemed a key factor linked to the nurses’ job satisfaction, which has been reported in previous studies as well [45]. Importantly, they felt very confident regarding their competencies to perform and to be efficient when caring for patients, which in turn, contributed to a sense of control in critical and high-stress situations that characterize much of the IC work. The sense of accomplishment and performance success, in turn, provided the nurses with feelings of being capable (i.e., self-efficacy) [109]. Self-efficacy has been found to be relevant both for older workers’ motivation at work, for their motivation to work [110], as well as for nurses’ willingness to work longer [44]. Self-efficacy and self-esteem are considered personal resources [111] that may protect and buffer the negative effects of heavy workload on nurses’ burnout. Thus, to promote aging-in-workplace and encourage extended working lives, healthcare organizations would need to develop practices that promote the development of nurses’ personal resources, including their occupational self-efficacy. Here, the possibilities for continuous learning and development that the IC unit provided, something that the nurses valued, may play a key role in developing personal resources, as this gave them opportunities to prevent skills obsolescence and to keep up with the rapid technological advances in the highly specialized environment of the IC unit.

### 4.11. Methodological Considerations and Suggestions for Future Research

The present study used a relatively small and homogenous sample of 12 nurses working in the same IC unit, which restricts generalizability to other organizations and nursing occupations while also restricting representativity. Yet, IPA studies often include smaller samples (between 5 and 10 participants) [58], which allow for keeping with the ideographic focus characterizing this approach [57]. The use of the IPA in this study involved a detailed and systematic analysis of the subjective experiences of working as an IC nurse, the perceptions of aging in the workplace, and thoughts regarding extended working lives in a particular healthcare setting in Sweden. The nurses that were interviewed had a good health and were very satisfied with their job and the organization, which means that there is a risk of sample selection issues resulting from a potential “healthy worker effect” [112]. This means that nurses with poorer health or those dissatisfied with their job or the workplace may have already left nursing or decided to not participate in this study. Moreover, the data were collected before the COVID-19 pandemic. Although most of the findings would stand in the present, the pandemic put an enormous pressure on IC healthcare workers who had to deal with an unknown, life-threatening disease, while the units also had to accommodate new and less experienced nursing staff in a very short time in order to respond to the higher IC needs [56]. Future research should thus investigate how the COVID-19 pandemic affected older IC nurses’ experiences of work and their late-career decisions. Additionally, interview studies with IC nurses in less supportive organizational contexts and in settings with more rigid pension systems, including nurses at different stages of their careers and specializing in different nursing occupations, may broaden the overall understanding of the topic of sustainable working lives in nursing.

## 5. Conclusions

The COVID-19 pandemic has put an enormous strain on the already pressured healthcare services all over the world [1]. Specifically, the health crisis brought about by the pandemic increased the demand for qualified healthcare staff and underscored the central role of nurses [1]. The characteristics of IC work have specifics that turn it into a particularly demanding environment—one with the highest staffing shortages in healthcare. Using a phenomenological approach, this study sought to add a fine-grained analysis to the research literature but also to provide policymakers and healthcare organizations with insights regarding IC nurses’ ways of conceptualizing and making sense of their experiences of work as well as their late-career decisions when approaching retirement, topics which seem understudied.

The IC nurses participating in this study planned to remain in the nursing profession and in the organization, and considered working until 65 and beyond. To work after retirement on an hourly basis was considered an attractive employment form in that it allows for flexibility in working time and more control over the type of tasks to be performed while simultaneously including a gradual disengagement from the working role, which in turn, can facilitate adapting to life as a retiree. The flexibility of the Swedish pension system, the labor demand for qualified nurses, and the openness of the organization to employ retired nurses were considered important contextual facilitators. While some of the emerging themes that resulted from the analysis follow existing findings from large quantitative studies with heterogeneous samples of older employees, the access to detailed and rich first-person accounts collected through the interviews with IC nurses served as a means to add the “lived experiences” of a specific profession and to provide a constellation of other individual, work, and organizational factors that seem central for ability and willingness to remain. Moreover, by combining an inductive and data-driven approach to identify themes emerging from the narratives with a deductive approach, informed by the SwAge model [59], to refine the thematic structure, this study also contributed to empirical validation of this model and to the research literature regarding sustainable working lives.

In terms of implications of the study, an improved focus on ergonomics, possibilities to adjust some tasks to age-related physical limitations and interests, a possibility for lateral job movement, and a formal mentoring program taking advantage of older nurses’ expertise while at the same time freeing them from some of the heavier tasks, were considered as examples of human resources management practices that would contribute to aging-in-workplace and increase the motivation to postpone the moment of fully exiting the nursing profession. Importantly, the findings from this study show that raising statutory retirement ages and establishing financial benefits for late retirement may constitute incentives for extended working lives; however, such incentives seem far from enough in order to encourage older nurses to postpone the timing of their exit from the labor market. Notably, it is important to ensure that older nurses want to, and will be able to, work longer, which is something that may be facilitated by providing adequate working conditions that enable a healthy and sustainable working life throughout the life-span.

## Figures and Tables

**Table 1:** Thematic structure.

Superordinate Themes	Subordinate Themes
1. Retirement decision making	1.1. Ambivalent attitudes towards retirement
	1.2. Retirement preferences
	1.3. The transition to retirement
2. Self-rated health, diagnoses, and functional diversity	2.1. Being healthy as a condition to continue working
	2.2. Being healthy as a condition to enjoy retirement
3. Physical work environment	3.1. Continuous noise causes stress
	3.2. A physically demanding job
4. Mental work environment	4.1. Being in control despite the unpredictable context
	4.2. Job demands—the “ethical stress” and the “nursing dilemma”
	4.3. Job demands also perceived as positive challenges
5. Work schedule, work pace, and recovery time	5.1. Irregular working time may be a hinder for a sustainable and healthy working life
	5.2. Recovery strategies
	5.3. Post-retirement work as a way to work in a flexible schedule
6. Personal finances	6.1. Financial considerations
7. Personal social environment and private life	7.1. Attitudes towards leisure
	7.2. Family situation plays a role in the work centrality and retirement plans
8. Social work environment, discrimination, leadership style, and age management	8.1. Organization-based self-esteem
	8.2. The importance of a supportive working team
	8.3. A supportive leadership is central for job retention
	8.4. Age-friendly work environment
	8.5. Age management practices that may promote a more sustainable working life
9. Motivation, satisfaction, and stimulation in the execution of work tasks	9.1. A rewarding and meaningful job
	9.2. Variety at work
	9.3. Job satisfaction
10. Competence, use of skills, knowledge, and opportunities for development at work	10.1. Opportunities for use of accumulated knowledge and competences
	10.2. Occupational self-efficacy and the positive side of being an older worker— “the clinical eye” and “the tacit knowledge”
	10.3. Importance of keeping skills up-to-date
	10.4. Internal and external employability

## Data Availability

The data are not publicly available due to legal restrictions that protect the integrity of research participants. Detailed analyses of the masked transcripts are available on request from the corresponding author [M.S.-R.].
